# Slc38a1 Conveys Astroglia-Derived Glutamine into GABAergic Interneurons for Neurotransmitter GABA Synthesis

**DOI:** 10.3390/cells9071686

**Published:** 2020-07-13

**Authors:** Tayyaba Qureshi, Mona Bjørkmo, Kaja Nordengen, Vidar Gundersen, Tor Paaske Utheim, Leiv Otto Watne, Jon Storm-Mathisen, Bjørnar Hassel, Farrukh Abbas Chaudhry

**Affiliations:** 1 Department of Molecular Medicine, Institute of Basic Medical Sciences, University of Oslo, 0317 Oslo, Norway; tayyabaraheel80@gmail.com (T.Q.); mona.bjorkmo@gmail.com (M.B.); kaja.nordengen@gmail.com (K.N.); vidar.gundersen@medisin.uio.no (V.G.); jonsm@uio.no (J.S.-M.); 2 Department of Plastic and Reconstructive Surgery, Oslo University Hospital, 0424 Oslo, Norway; utheim2@gmail.com; 3 Department of Geriatric Medicine, Oslo University Hospital, 0424 Oslo, Norway; l.o.watne@medisin.uio.no; 4 Department of Neurohabilitation, Oslo University Hospital and University of Oslo, 0424 Oslo, Norway; bjornar.hassel@medisin.uio.no

**Keywords:** astrocyte-neuron shuttle, GABA, glutamate, glutamine transporter, interneuron, neurotransmitter replenishment, SNAT1, SAT1, Slc38, system A

## Abstract

GABA signaling is involved in a wide range of neuronal functions, such as synchronization of action potential firing, synaptic plasticity and neuronal development. Sustained GABA signaling requires efficient mechanisms for the replenishment of the neurotransmitter pool of GABA. The prevailing theory is that exocytotically released GABA may be transported into perisynaptic astroglia and converted to glutamine, which is then shuttled back to the neurons for resynthesis of GABA—i.e., the glutamate/GABA-glutamine (GGG) cycle. However, an unequivocal demonstration of astroglia-to-nerve terminal transport of glutamine and the contribution of astroglia-derived glutamine to neurotransmitter GABA synthesis is lacking. By genetic inactivation of the amino acid transporter Solute carrier 38 member a1 (Slc38a1)—which is enriched on parvalbumin^+^ GABAergic neurons—and by intraperitoneal injection of radiolabeled acetate (which is metabolized to glutamine in astroglial cells), we show that Slc38a1 mediates import of astroglia-derived glutamine into GABAergic neurons for synthesis of GABA. In brain slices, we demonstrate the role of Slc38a1 for the uptake of glutamine specifically into GABAergic nerve terminals for the synthesis of GABA depending on demand and glutamine supply. Thus, while leaving room for other pathways, our study demonstrates a key role of Slc38a1 for newly formed GABA, in harmony with the existence of a GGG cycle.

## 1. Introduction

GABA, the principal fast inhibitory neurotransmitter in the central nervous system (CNS), is pivotal to regulate neuronal excitability and function [1]. Classical GABA signaling depends on the quantal release of this neurotransmitter from nerve terminals, activation of postsynaptic GABA_A_ receptors and termination of synaptic neurotransmission by removal of GABA from the synaptic cleft. Such short-lived and phasic signaling regulates action potential firing, synchronizes ensembles of neuronal networks and controls the efficacy and plasticity of excitatory inputs onto principal neurons. GAT1, a plasma membrane GABA transporter enriched on nerve terminals, may sequester released GABA for reuse in synaptic neurotransmission [2]. However, there is also a substantial drain of GABA into perisynaptic astroglial processes [3]. Consequently, sustained GABA signaling requires efficient local means for neurotransmitter replenishment. Considering the importance of GABA signaling for brain functions and the detrimental consequences of its malfunction, such as in neurodegeneration, epilepsy and anxiety [4,5,6], it is surprising that rate-limiting mechanisms for GABA replenishment remain elusive.

The system N glutamine/amino acid transporter Slc38a3 (also known as (aka) SN1 and SNAT3) is localized on perisynaptic astroglial processes and readily releases glutamine [7,8,9]. As the homologous system A transporter Slc38a1 (aka SAT1, SA2 and SNAT1) is enriched on parvalbumin+ interneurons and preferentially transports glutamine [10,11], we hypothesized that Slc38a1 may be essential to replenish GABA and, thus, a significant regulator of synaptic plasticity. Indeed, we have demonstrated major roles of Slc38a1 in parvalbumin^+^ GABAergic interneurons [12]: Slc38a1 impacts on vesicular GABA load and synaptic vesicle dynamics and contributes to cortical processing and plasticity. When activated by the transport of glutamine it induces high-frequency membrane oscillations, which may trigger action potentials. Investigation of Slc38a1^−/−^ mice showed significantly reduced levels of glutamine, glutamate, GABA and aspartate in total brain lysates by high-performance liquid chromatography (HPLC), compared to Slc38a1^+/+^ mice, suggesting that Slc38a1 is pivotal for the synthesis of GABA. However, these investigations did not reveal the origin of the GABA formed. Here, we investigated whether GABA synthesis in GABAergic interneurons depends on astroglia-derived glutamine, using radiolabeled acetate, and evaluated contribution of Slc38a1 for de novo synthesis of GABA in nerve endings of GABAergic interneurons. 

## 2. Materials and Methods

### 2.1. Animal Handling

Experiments were approved and conducted in accordance with the Norwegian Animal Welfare Act (No. FOTS 91009/date of approval: the 27 of August 2019) and the European Convention for the Protection of Vertebrate Animals used for Experimental and Other Scientific Purposes (ETS 123). The mice were kept and handled under veterinary supervision at the UiO. Mice were housed in a temperature controlled facility (22–26 °C) with 50 ± 10% humidity and a 12 h light/dark cycle. Animals were served municipal water and fed ad libitum with RM3 (E) from Special Diets Services (UK).

### 2.2. Labeling Experiments with ^14^C- and ^13^C-Labeled Acetate

Mice (five Slc38a1^+/+^ and Slc38a1^−/−^ pairs) received 10 μL/g body weight i.p. of sodium [1-^13^C]acetate, 300 µmol/mL, with [2-^14^C]acetate, 60 µCi/mL (final specific activity 0.2 µCi/µmol). At 10 min the animals were killed by cervical dislocation and immediately decapitated, dropping the heads into liquid N_2_. Perchloric acid extracts of brains and sera were prepared and freeze-dried [13]. Forebrain samples were redissolved in 250 μL of distilled water with α-aminoadipate, 1 mmol/L, as an internal concentration standard. Amino acids were separated and quantified by HPLC and fluorescence detection after precolumn derivatization with o-phthaldialdehyde [13]. The HPLC eluate was collected in 1 min fractions over 60 min, a process which gave an excellent separation of amino acids, and the radioactive labeling of individual amino acids was analyzed by scintillation counting [14]. Serum samples were redissolved in 400 μL of D_2_O with dioxan, 0.1% (*v*/*v*), as internal concentration standard, and analyzed by ^13^C nuclear magnetic resonance spectroscopy, as described elsewhere [13].

To monitor isotopic labeling of compounds in serum by NMR spectroscopy, [^14^C]acetate was injected i.p. together with [^13^C]acetate. Only ^13^C-labeled acetate and no ^13^C-labeled glucose or lactate was seen in serum (data not shown), ascertaining that the labeling of amino acids in the brain was from [^14^C]acetate taken up by the brain and not from a circulating ^14^C-labeled metabolite of acetate. 

### 2.3. Preparation and Incubation of In Vitro Hippocampal Slices

Hippocampal slices were prepared largely as previously described [15]. Slc38a1^+/+^ and Slc38a1^−/−^ mice were decapitated and the brains were put in ice-cold normal Krebs’ solution (in mM: 130 NaCl, 3 KCl, 15 sodium phosphate buffer, 1.2 CaCl_2_, 1.2 MgSO_4_, and 10 glucose). Hippocampi were sliced (300 μm) and incubated for 45 min at 30 °C (after preincubations in normal solution without glutamine at 30 °C for 45 min) in oxygenated (100% O_2_) normal (3 mm K^+^) or depolarizing (50 mm K^+^; Na^+^ reduced to 83 mm) Krebs’ solutions with or without the presence of glutamine (0.5 mM, i.e., close to the physiological level in brain extracellular fluid). The slices were kept on a nylon mesh in a glass beaker with a continuous flow of O_2_ over the surface of the medium. After incubation, the slices were fixed for 1 h at room temperature (∼22 °C) in a mixture of 1% formaldehyde and 2.5% glutaraldehyde in 0.1 M sodium phosphate buffer pH 7.4. The slices were kept overnight at 4 °C in the same fixative, and stored in the fixative diluted 1:10.

### 2.4. Immunoperoxidase Processing and Analyses of Rat Brain Slices

The specificity of the antibody (990 GABA) towards GABA has previously been optimized and extensively tested [16,17]: It was first purified on an agarose column containing glutamate coupled to bovine serum albumin by glutaraldehyde. Then, ~50 different amino acids and endogenous peptides of the brain, fixed to brain total macromolecules by glutaraldehyde, were deposited on cellulose nitrate–acetate filters and the purified 990 GABA antibody tested on them. Only some minor cross-reactivity had remained towards L-glutamate and β-alanine. This cross-reactivity vanished upon preabsorption of the antibody preparation with glutaraldehyde/formaldehyde complexes of glutamate and β-alanine. The brain slices were stained for GABA by preabsorbed 990 GABA antibody for immunocytochemistry using the biotin-streptavidin-peroxidase system and 3,3′-diaminobenzidine (DAB) as chromogen, as described elsewhere [18]. A spot-test, prepared as described [16,19] was included.

The slices were examined by transmitted light microscopy, including differential interference contrast (DIC). For quantitative analyses, ordinary bright field microphotographs were taken with a 20× objective, at fixed Koehler illumination intensity and exposure time. Absorbance (A, i.e., optical density, reflecting the approximate concentration of GABA) was determined by ImageJ (1.52a) software (https://imagej.nih.gov/ij/), measuring grayvalues in squares 20 µm × 20 µm placed nonoverlappingly over the pyramidal cell layer-to-oriens layer interface (which has the highest density of GABAergic terminals). Before analysis, the tiff images were converted in ImageJ to monochrome, 8-bit pixel depth, i.e., 256 grayvalues. A was calculated by the formula A = −log_10_T = log_10_ I_0_/I, where I and I_0_ are the intensities of transmitted and incident light, respectively. ImageJ quantifies T by grayvalues, where 0 is black (all light absorbed) and 255 is 100% transmission of light. I_0_ is the grayvalue measured outside the tissue specimen (slightly less than 255 due to light absorption in glass and mounting medium) and I is the grayvalue of the individual pixels in the area observed. The ImageJ function “Measure” delivers the minimum, maximum, and mean (average) grayvalue of the pixels in the area measured, i.e., the darkest (T_min_ converted to A_max_), lightest (T_max_ converted to A_min_) and mean (T_mean_ converted to A_mean_) pixel value. Net A_mean_, reflecting the mean GABA concentration in the measured area, was obtained for each measuring area in each slice category after subtraction of A_mean_ measured over a slice processed without primary antibody, i.e., representing absorption of light in tissue without a GABA signal. Net A_mean_ results from two experimentsare presented, as percent of the average net A_mean_ value in Slc38a1^+/+^ mice (wt) without glutamine supplementation, of 6 measured squares for each category. Densitometric immunoperoxidase quantification, as performed here, is known to be able to produce results with high fidelity, shown, e.g., for tyrosine hydroxylase in the locus coeruleus, where the absorbance of immunocytochemically formed DAB polymer correlated linearly with biochemically measured enzyme activity at *r* > 0.9 [20].

In complementary analyses, correction for the background was obtained by subtracting pixel values of A_min_ from A_max_. As both contain “background” absorption of light by tissue, unrelated to GABA content, this cancels out in the subtraction. A_max_ reflects the GABA concentration in the nerve endings that are the highest in GABA, whereas A_min_ reflects (low) GABA signal in other structures (with some contribution of signal shining through from GABAergic terminals out of focus). The highest readings of A_max_ (0.6421 and 0.8665 in Experiments 1 and 2, respectively) corresponded to about 300% of the mean net absorbance values for Slc38a1^+/+^ mice (wt) without glutamine supplementation (100%). This indicates that saturation of the signal (above which absorbance does not increase with an increase in chromogen concentration) did not occur in the range observed and therefore is unlikely to have affected the conclusions.

Finally, numbers of GABA^+^ “puncta” (representing nerve endings and axon branches) were counted, blind, in 12 rectangular frames 5 µm × 20 µm distributed randomly and nonoverlappingly over the pyramidal cell layer and the immediately subjacent part of the oriens layer of hippocampus CA1, and presented as mean ± SEM. 

Statistical significance was estimated by Student’s *t*-test, two tails.

## 3. Results

We investigated the (in-)ability of Slc38a1^−/−^ mice to synthesize GABA de novo. Radiolabeled acetate, an astroglia-specific energy substrate, which selectively radiolabels astroglial glutamate and glutamine (for a review, see [21]), was injected i.p., and radiolabeling of amino acids was determined by scintillation counting after separation by HPLC. 

**Figure 1:**
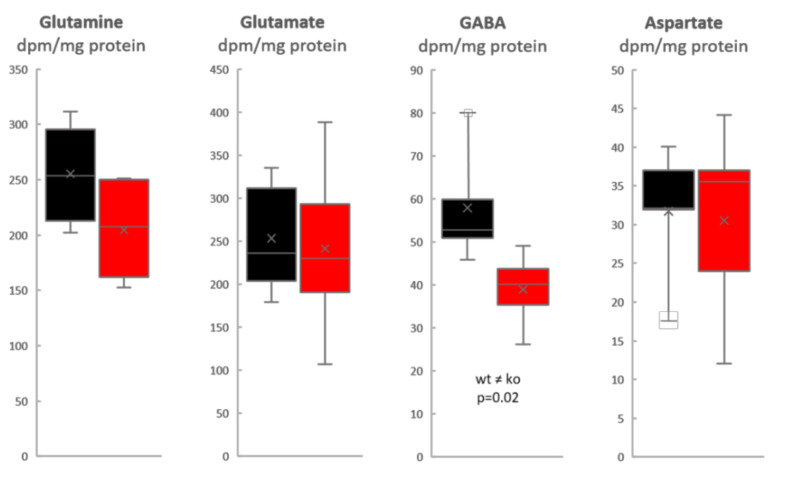
Significant reduction in the newly formed GABA from the astroglia-specific metabolite acetate in Slc38a1^−/−^ mice. Slc38a1^+/+^ and Slc38a1^−/−^ mice were given an intraperitoneal injection of [2-^14^C]acetate and sacrificed 10 min after injection to assess the capability of Slc38a1^−/−^ (red) and Slc38a1^+/+^ (black) mice to synthesize glutamine, glutamate, GABA and aspartate. Values are given as radioactivity/mg protein to show changes in newly made amino acids. Glutamine, glutamate and aspartate are made to the same degree in both genotypes. In contrast, there is a significant reduction in the newly synthesized GABA in Slc38a1^−/−^ (*p* = 0.02, two-sided paired Student’s *t*-test). Data are presented as box-and-whisker plots (Excel): whisker is range (highest and lowest observed value), × is mean (average), – is median, box is the interquartile range (middle 50% of the observed values); n = 4 mice of each genotype for GABA and glutamine, n = 5 mice of each genotype for glutamate and aspartate.

**Figure 2:**
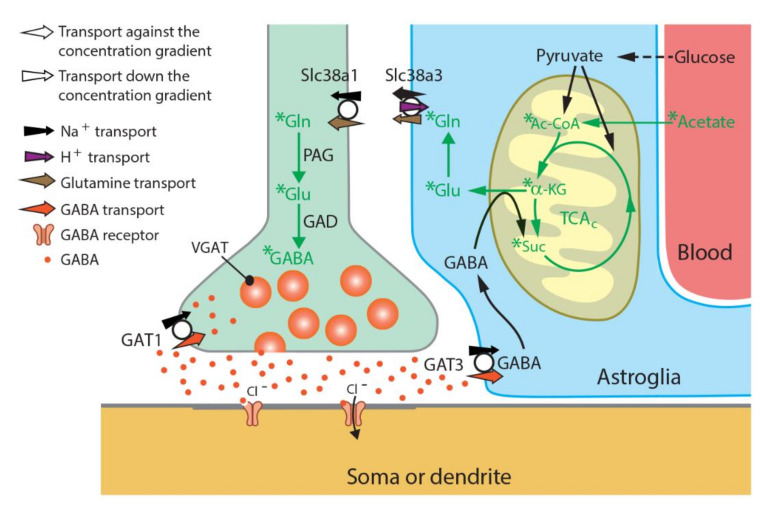
Schematic representation of how acetate contributes to glutamine and GABA synthesis at GABAergic synapses in normal physiological conditions. Radiolabeled acetate, injected intraperitoneally, approaches brain tissue by blood flow and is selectively metabolized in astroglial cells to form glutamine. Our findings (Figure 1) on labeled glutamine, glutamate and GABA together with their relative ratios in Slc38a1^+/+^ mice suggest that glutamine formed in astroglia is shuttled to the GABAergic neurons by the glutamine transporters Slc38a3 and Slc38a5 (also known as (aka) SN1 and SNAT3, and SN2 and SNAT5, respectively) and Slc38a1 (aka SAT1, SA2 and SNAT1) at astroglial and neuronal cell membranes, respectively, and sustains GABA pools. Genetic inactivation of Slc38a1 results in significantly lower levels of radiolabeled GABA, implicating that astroglia-derived glutamine and its transport through Slc38a1 is essential for GABA formation and bolsters the existence of a glutamate/GABA-glutamine (GGG) cycle. TCA_C_, tricarboxylic acid cycle; Ac-CoA, acetyl coenzyme A; α-KG, α-ketoglutaric acid; Suc, succinate; PAG, phosphate-activated glutaminase; GAD, glutamic acid decarboxylase; GAT1/3, GABA transporter 1/3; VGAT, vesicular GABA transporter; Gln, glutamine; Glu, glutamate; GABA, γ-aminobutyric acid.

We find that the specific activity of glutamine (expressed as radioactivity dpm/nmol amino acid) was approximately 3× higher than that of glutamate in both Slc38a1^+/+^ and Slc38a1^−/−^ mice: the glutamine/glutamate specific activity ratio was 2.9 ± 0.7 and 2.9 ± 0.6 (mean ± SD) in Slc38a1^+/+^ and Slc38a1^−/−^ mice, respectively, supporting acetate utilization by astrocytes, which unlike neurons express glutamine synthetase [22]. The specific activity of glutamine was approximately twice that of GABA: the glutamine/GABA specific activity ratio was 2.2 ± 0.6 and 2.7 ± 0.7 (mean ± SD) in Slc38a1^+/+^ and Slc38a1^−/−^ mice, respectively, which agrees with a precursor-product relationship for glutamine (as precursor) and GABA (as product). The radiolabeling (specific activity) of glutamine and that of GABA were strongly correlated when data for Slc38a1^+/+^ and Slc38a1^−/−^ mice were analyzed together (*r* = 0.91; *p* = 0.0003; Pearson product–moment correlation). The formation of radiolabeled GABA from [^14^C]acetate is significantly reduced in Slc38a1^−/−^ mice (Figure 1—data given as radioactivity/mg protein to focus on newly formed amino acids), implicating a role for Slc38a1 in accumulating glutamine in GABAergic neurons for GABA synthesis. However, the data indicate that this takes place in a subpopulation of GABAergic neurons and/or support the existence of alternative mechanisms of GABA replenishment as the reduction in GABA formation is moderate (mean value reduced by one third, Figure 1). More importantly, as the labeled precursor is made from labeled acetate in astroglia, the data ascertain astroglia-to-neuron shuttling of glutamine and thereby the existence of a GGG cycle (Figure 2).

**Figure 3:**
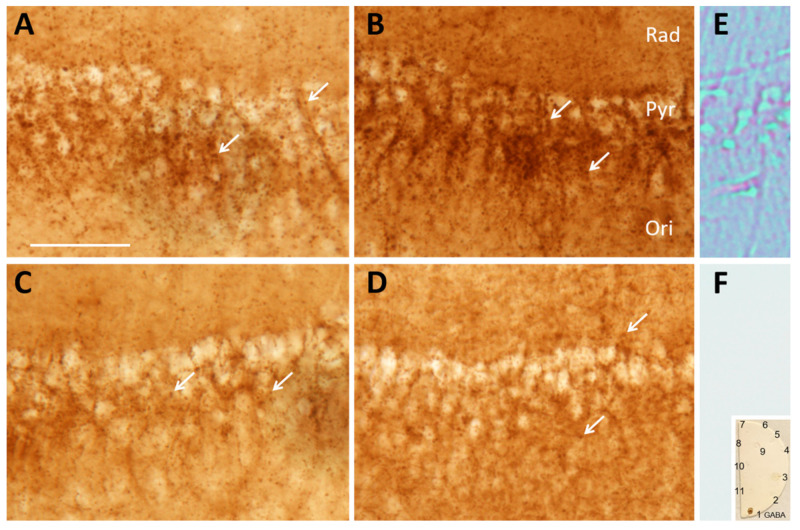
GABA synthesis in nerve endings is rescued by glutamine supplementation in Slc38a1^+/+^ mice, but not in Slc38a1^−/−^ mice. Hippocampal slices from Slc38a1^+/+^ (wt) and Slc38a1^−/−^ (knock-out (ko)) mice were depolarized by 55 mM K^+^ for 45 min in the absence or presence of glutamine, and then aldehyde fixed and stained for GABA by the immunoperoxidase method. Microphotographs from representative slices, taken at constant bright field illumination and constant exposure time, show GABA-immunoreactivity in region CA1 of hippocampal slices from wt (**A**,**B**) and ko (**C**,**D**) male mice, incubated with glutamine (**B**,**D**), or without glutamine (**A**,**C**). Note “puncta”, representing GABA-containing nerve endings (*arrows*), adjacent to unstained pyramidal cell bodies. Staining intensity is increased by glutamine in wt (**B**), but not in ko (**D**), see quantification in Figure 4. Sections processed without antibody to GABA (**E**,**F**) serve to estimate “background” absorption of light in unstained tissue, without a GABA signal. They show no peroxidase staining (**F**), only non-Koehler illumination revealed refraction at membranes of nerve cells and vessels (**E**). A test-“section” (F, *inset*), incubated as tissue sections and carrying spots of amino acids glutaraldehyde fixed to brain macromolecules, shows excellent specificity for GABA. (Amino acids in spots: 1 GABA, 2 none, 3 glycine, 4 D-serine, 5 L-cysteine, 6 D-aspartate, 7 L-serine, 8 L-valine, 9 D-cysteine, 10 L-glutamate, 11 L-α-alanine.) Rad, stratum radiatum; Pyr, stratum pyramidale; Ori, stratum oriens. Scale bar = 50 µm.

To further explore the ability of inhibitory nerve terminals to synthesize GABA and the dependence of this on Slc38a1-mediated glutamine transport, we depolarized hippocampal slices from Slc38a1^+/+^ and Slc38a1^−/−^ mice with 55 mM K^+^ to deplete neurotransmitter reservoirs and stimulate replenishing biogenesis [23]. Subsequent immunolabeling for GABA showed punctate GABA-like immunostaining around hippocampal pyramidal cell bodies and in the adjacent layers radiatum and oriens, consistent with the localization of perisomatic, peridendritic and (in oriens) periaxonal GABA-containing terminals (cf., [24]). The staining intensity in Slc38a1^+/+^ mice (Figure 3A) appeared higher than in Slc38a1^−/−^ mice (Figure 3C). In similar conditions, but during supplementation with glutamine at physiological extracellular concentration (0.5 mM), the staining intensity for GABA increased considerably in Slc38a1^+/+^ mice (Figure 3B). No such difference was found in slices from Slc38a1^−/−^ mice (Figure 3D).

**Figure 4:**
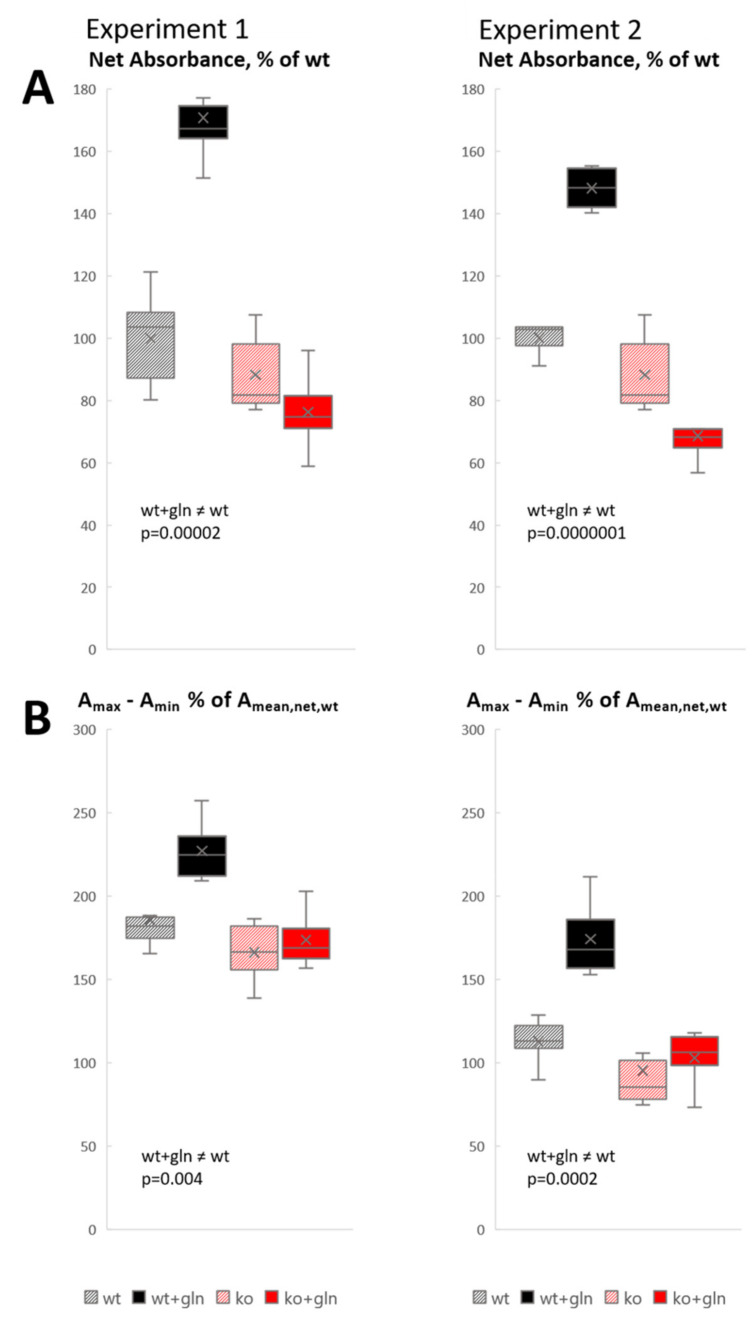
GABA synthesis in nerve endings is rescued by glutamine supplementation in Slc38a1^+/+^ mice, but not in Slc38a1^−/−^ mice: densitometric analyses. Hippocampal slices from Slc38a1^+/+^ (wt) and Slc38a1^−/−^ (ko) mice were treated with and without glutamine (gln) as indicated in Figure 3. Data from two experiments are shown as whisker-and-box plots (Excel): whisker is range (highest and lowest observed values), × is mean, − is median, box is the interquartile range (middle 50% of values). (**A**) Net absorbance (A, representing approximate GABA concentration), measured in 6 squares 20 µm × 20 µm at the Pyr/Ori interface, expressed as % of that in wt. Corresponding “background” readings, in sections processed without GABA antibody (Figure 3F) to measure light absorption in tissue without GABA signal, were subtracted to obtain net GABA signal. (The subtracted “background” absorbance 0.0258 ± 0.0005 (mean ± SD) corresponded to 13% of the net wt absorbance in experiment 1, 8% in Experiment 2) (**B**) In each measured 20 µm × 20 µm square, the absorbance in the darkest pixel, A_max_, and in the lightest pixel, A_min_, were subtracted, canceling out “background”, and difference expressed as % of the net mean absorbance in wt (**A**). This estimates how much the GABA signal in the nerve endings with the highest GABA levels exceeds the “general” GABA signal in tissue elements with low GABA content (with some addition of signal from GABA-containing structures that are out of focus in the measured field).

The visual observations were confirmed by quantitative analysis of absorbance (optical density), reflecting the tissue GABA concentration (Figure 4). Two experiments showed consistent results (Figure 4, left and right). Measurements at the pyramidal cell layer-to-oriens layer interface (which has the highest density of GABAergic terminals, Figure 3A) showed a highly significant increase of GABA signal on glutamine supplementation in Slc38a1^+/+^ mice, but not in Slc38a1^−/−^ mice. Without glutamine, there was a tendency towards lower GABA signal in Slc38a1^−/−^ mice compared to Slc38a1^+/+^ mice, but this did not reach statistical significance. In Figure 4B, we contrasted the darkest pixel in each measuring field (i.e., the pixel with the highest absorbance, A_max_) with the lightest (i.e., the lowest, A_min_). The darkest pixel represents the nerve ending with the highest GABA concentration. The lightest pixel represents a site where no GABA-containing nerve ending is in focus, color being due to slight GABA signal in other tissue elements, with some contribution from GABA-stained structures that are out of focus. (Both the darkest and the lightest pixel has a “background” that is not due to GABA, but to light absorption by unstained tissue. This background cancels out when the two values are subtracted.) The difference A_max_ − A_min_ estimates to what extent the GABA level in the nerve endings richest in GABA exceeds a general tissue level. The results show that in slices from Slc38a1^+/+^ mice, this difference is significantly higher with glutamine than without glutamine, indicating that the glutamine effect occurs in the individual GABAergic terminal. No statistically significant differences were found among the other categories. The magnitude of the A_max_ − A_min_ differences considerably exceeds the average total GABA level (Figure 4A), attesting to the enrichment of GABA in GABAergic terminals.

The density of GABA-stained “puncta” (representing GABA^+^ nerve terminals and axons), counted blind in 12 random nonoverlapping 5 µm × 20 µm frames at the pyramidal cell layer-to-oriens layer interface, increased slightly with glutamine supplementation in Slc38a1^+/+^ mice (Figure 3B versus A; 21.20 ± 0.63 versus 19.25 ± 0.70, mean ± SEM, *p* = 0.04), indicating that some terminals with low GABA levels were brought above the detection limit by glutamine. 

The inability of extracellular glutamine to rescue GABA production in Slc38a1^−/−^ synaptic terminals supports the notion that Slc38a1-mediated glutamine uptake is a primary source of newly formed GABA for synaptic neurotransmission. Altogether, our data demonstrate that Slc38a1 is required for glutamine supplementation to augment GABA synthesis. 

## 4. Discussion

### Slc38a1 Furnishes GABAergic Neurons with Glutamine to Synthesize Neurotransmitter GABA De Novo

We demonstrate that GABA synthesis in GABAergic nerve terminals in brain slices obtained from Slc38a1^+/+^ mice increases upon depolarization in the presence of glutamine. In contrast, glutamine supplementation fails to augment GABA synthesis under similar conditions in brain slices from Slc38a1^−/−^ mice. These data bolster the role of Slc38a1 in glutamine uptake into GABAergic nerve terminals for the replenishment of the neurotransmitter GABA. These data also lend support to our previous findings, in GABAergic neurons of Slc38a1^−/−^ mice, on selective and significant reduction of GABA and/or amino acids involved in GABA metabolism (i.e., glutamine, glutamate and aspartate) by HPLC analyses of whole-brain homogenates and synaptosomal fractions and by immunogold electron microscopy [12]. These data are also consistent with the reported upregulation of phosphate-activated glutaminase (PAG) and glutamic acid decarboxylase 67 (GAD67) as a compensatory action to restore the neurotransmitter GABA levels in Slc38a1^−/−^ mice [12]. Our data thus agree with the finding that system A activity is responsible for 87% of neuronal uptake of glutamine [25] and that system A mediated glutamine uptake maintains GABA synthesis and sustains inhibitory synaptic transmission [26]. Indeed, a large number of publications show that glutamine can maintain GABA levels in GABAergic nerve endings [27,28,29]. Our study identifies Slc38a1 as the transporter responsible for this effect. Our data also show that the GABAergic nerve terminals in hippocampus CA1 have some residual GABA immunostaining in Slc38a1^−/−^ mice (both with and without glutamine). This observation is consistent with Slc38a1 primarily being enriched in the nerve terminals of parvalbumin^+^ interneurons and therefore Slc38a1 inactivation only affects this compartment in this subpopulation of GABAergic neurons [12]. It also suggests that additional mechanisms exist for GABA synthesis in parvalbumin^+^ neurons in the Slc38a1^−/−^ mice. Transfer of astroglial metabolites other than glutamine—such as α-ketoglutarate, malate and succinate—to sustain neuronal anaplerosis and GABA synthesis has also been demonstrated (for review, see [30]. There is also evidence for the existence of other glutamine transporters yet to be molecularly identified [31]. System ASC and system L glutamine transporters are localized in brain tissue and may also contribute to GABA synthesis (e.g., [32]. Finally, Slc38a2 is sub-significantly increased in Slc38a1^−/−^ mice, and may also contribute to GABA synthesis [12]. In addition, expression of PAG and GAD67 is compensatorily increased in the Slc38a1^−/−^ mice to boost GABA synthesis and counteract its depletion [12]. Thus, there are several important alternative pathways for glutamine transport and GABA synthesis which may partially rescue the phenotype. However, Slc38a1 is a key component of a functional GGG cycle at GABAergic neurons.

The hypothesis of a GGG cycle, proposed decades ago, postulates that GABA (and glutamate) upon transport into astroglial cells is converted to glutamine, which is transported back into neurons for the regeneration of the neurotransmitter. This hypothesis is supported by (1) plasma membrane GABA (and glutamate) transporters being localized on perisynaptic astroglial processes [3,33], (2) glutamine synthetase (GS), the only enzyme catalyzing the formation of glutamine, being selectively expressed by astroglial cells [34] and (3) phosphate-activated glutaminase (PAG), catalyzing the transformation of glutamine to glutamate, being enriched in nerve terminals [35,36] (for a review, see [37]. Subsequently, glutamate may be converted to GABA in neurons expressing glutamic acid decarboxylase 65 or 67 isoforms (GAD65 or GAD67) [4]. However, a role for glutamine in GABA replenishment and the existence of a GGG cycle have been difficult to demonstrate because experimental evidence for an astroglia-to-neuron glutamine shuttle has been lacking. The existence of a GGG cycle has even been challenged by studies showing preserved neurotransmission upon removal of external glutamine or inhibition of GS or system A activity [38]. A role of system A transporters for neurotransmitter replenishment was dismissed in another study, reporting that the glutamate–glutamine cycle was inhibited by histidine and not MeAIB, the prototypic substrate of system A activity [31]. ^14^C-Acetate, used in our study, is selectively metabolized by astrocytes and converted into ^14^C-labeled glutamine. Thus, the formation of ^14^C-labeled GABA relies on the release of glutamine from astrocytes and uptake into GABAergic neurons, a process that is significantly reduced in the Slc38a1^-/-^ mice. Our data therefore demonstrate a functional and metabolic coupling between astroglial cells and neurons and that the system A transporter Slc38a1 is important for neuronal glutamine uptake for neurotransmitter GABA synthesis according to the GGG cycle. Indeed, our data harmonize with a large number of reports indicating that astroglial cells are the principal source of glutamine for GABA synthesis [26,39,40,41,42]. Consequently, impairment of the astroglial GS results in reduced inhibitory synaptic activity and/or neuronal dysfunction [43,44,45]. 

The elucidation of cell-specific localization and peculiar kinetics of the members of the Slc38 family of glutamine/amino acid transporters provides both novel insights into cellular glutamine transport in the CNS and the complexity of GABA recycling [46]. Slc38a3 and Slc38a5 (aka SN2 or SNAT5) reside on astroglial membranes and work bi-directionally to supply neurons with glutamine [7,8,47,48]. The homologous unidirectional system A transporter Slc38a2 (aka SAT2, SA1 and SNAT2) accumulates glutamine in the dendrites of glutamatergic neurons to form glutamate used in retrograde signaling [18]. Slc38a1 is also a unidirectional system A transporter, which transports glutamine readily [10,49]. The present demonstration of de novo synthesis of GABA from astroglia-derived glutamine and Slc38a1-dependent glutamine uptake into GABAergic nerve terminals further reveals the importance of this family of transporters for neurotransmitter replenishment. Indeed, the hippocampal nerve terminals with increased Slc38a1-dependent GABA staining upon glutamine supplementation are surrounded by astroglial processes with the highest staining for Slc38a3 [50,51] further supporting the involvement of Slc38 transporters in GGG cycling.

GABA signaling undergird learning, memory, cognition and sensory perceptions [52], and perturbed GABA signaling contributes to brain diseases, such as neurodegeneration, epilepsy, anxiety and depression [6,53,54,55,56]. Slc38a1 itself is targeted by β-amyloid and other molecules leading to loss of the protein ([57], Chaudhry et al., unpublished data). Consequently, Slc38a1 may be involved in pathophysiology and may also have potential as a therapeutic target.
